# Supplementary material for: Evaluating profitability of beef cattle farming and its determinants among smallholder beef cattle farmers in the Baljovan District of Khatlon region, Tajikistan
Source: PLoS One. 2022 Sep 13;17(9):e0274391. doi: 10.1371/journal.pone.0274391 (PMC9469962; doi:10.1371/journal.pone.0274391)
Supplement: S1 File — (PDF) [file pone.0274391.s001.pdf]

S1. A household questionnaire.

Dear respondents.

Farrukh Jobirov is a PhD student at Jilin Agricultural University in China, where I am pursuing a doctorate in Agricultural Economics and Management. I'm embarking on a study entitled "Evaluating Profitability of Beef Cattle Farming and its Determinants among Smallholder Beef Cattle Farmers in the Baljovan District of Khatlon Region, Tajikistan." I gladly request your cooperation and assistance in responding to the questionnaire's questions. The information received will be used exclusively for academic reasons and will be kept strictly secret. No personal information will be made available to the public.

Thank you very much for your support.

Best regards

Farrukh Jobirov

Questionnaire on Profitability of Beef Cattle Farming and its Determinants among Smallholder Beef Cattle Farmers in the Baljovan District of Khatlon Region, Tajikistan.

Region………….**District**……………..**Ward**…………..**Village...........Respondent's Names**…………………**Phone number**…………………..

SECTION A: Background Information

1. Household head's sex 1) male 2) female
2. Age……………..years
3. Marital status 1) single 2) married 3) Divorced 4) widowed
4. Education level………………….years and (Tick where appropriate).
   1=No formal education [ ] 2=Primary education [ ] 3= Secondary education [ ] 4=College education [ ]
5. How many family members (family size) in the household …………………..?

SECTION B: Household Enterprises (Livestock production information)

6. What livestock types and numbers do you have on your farm?

**Livestock type**	Is it kept on the farm (farm house)? **(**Tick where applicable)		Average number kept in the last 12 months
	**Yes**	**No**	
**Cattle**			
**Sheeps**			
**Goats**			
**Pigs**			
**Poultryy**			
**Camels**			

**Donkey**			
**Other (specify)**			

7. Which activities does the household mainly depend on for livelihood, e.g., for provision of income, food, fees etc.?

**Enterprise**	Is it a source of Livelihood? (Tick where applicable)		If YES, please indicate the proportion of monthly income in an average year, from each enterprise which is a source of livelihood (*TICK one applicable range for each enterprise*)			
	**Yes**	**No**	*Less than quarter (<25%)*	*Between quarter and half (25-50%)*	*Between half and three quarters (50-75%)*	*More than three quarters (>75%)*
Beef cattle						
Other livestock (specify the mainone___________________)						
Crops (specify the main one______________________)						
Off-farm employment						
Others						

8. For how many years have you practiced beef cattle production? __________ years.
9. What is your main source of labour used in beef cattle production?
1= Family [ ] 2= Hired [ ] 3= both family and hired [ ]
10. How many days in a month are you normally available on the farm? ______ days.
11. Is there a manager on the farm, besides the household head?__ (1 = Yes, 2 = No).

SECTION C: Cattle Output

12**.** What is your current beef cattle herd size...?

13**.** Please provide information on beef cattle production in your farm. (Fill responses in the non-shaded blank areas)

	Calves		Heifers		Cows		Bulls		Steers(Oxen)	
	Now	*12 months ago*		*12 months ago*	*Now*	*12 months ago*	*Now*	*12 months ago*	*Now*	*12 months ago*
How many do you have (did you have)										
What is the average age of the beef cattle in the farm (years and months)										
		Calves in the last 12 months		*Heifers in the last 12 months*		*Cows in the last 12 months*		*Bulls in the last 12months*		*Steers (Oxen) in the last 12months*
How many did										

you purchase										
What was the average purchase price (US$)										
How many did you receive from other sources, e.g., dowry, gifts										
How many did you sell										
What was the average sales price (US$)										
How many did you use for other purposes e.g., consumption, gifts, dowry										

SECTION D: **Beef cattle Losses**

14**.** Have you had beef cattle die due to any of the following factors during the last 12 months? if YES, please indicate the number.

**Cause of loss**	Did beef cattle die from this cause? (Tick where applicable)		If **YES**, please indicate the number of beef cattle lost
	**Yes**	**No**	
Disease			
Drought			
Floods			
Landslides			
Thunder/lightning			
Disputes over pasture and water			
Attacks by wild animals			
Other factors (please specify)			

SECTION E: Variable Inputs

15**.** Please provide information on the inputs used in the beef cattle farm. (Should refer to periods within the last 12 months). Do NOT fill the shaded areas.

I**nputs**	**Average quantity used for all beef cattle per month**	**Total cost (US$) per month**
a) Purchased feeds		
Silage e.g., sunflower, rye, corn (Kilograms)		
Fodder e.g., hay, maize stalk/stover, wheat straw, sugarcane straw, rice straw, grass (Kilograms)		

Other feeds e.g., soyabean meal, urea (Kilograms)		
Mineral salt and vitamins (Kilograms)		
Water (litres)		
b) Feeds produced and used in the farm		
Silage e.g., sunflower, rye, corn (Kilograms)		
Fodder e.g., hay, maize stalk/stover, wheat straw, sugarcane straw, rice straw, grass (Kilograms)		
Other feeds e.g., soyabean meal, urea (Kilograms)		
Mineral salt and vitamins (Kilograms)		
Water (litres)		
c) Labour		
How many people are employed?		
How many days do the employed labourers per month?		
How much money is each farm employee paid per month?		
How many family members work?		
How many days do the family members work per month?		
How much money is each family member paid for working per month?		
How many unpaid people work?		
How many days do the unpaid people work per month?		
d) Cost of veterinary drugs and services		
e) Farm mechanization costs		
Fuel (litres)		
Electricity		
Hire of machinery and/or equipment, repairs and maintenance (per month)		
f) Other costs per month, e.g.,market services, ropes, branding, dehorning, etc.		

SECTION F: Fixed Inputs

16. Does the household own any of the following assets on the farm? if **YES**, please provide the following information. If **NO**, Go to Question 21. Do NOT fill the shaded areas.

Asset	Is it owned in the house hold? (1 = Yes 2 = No)	Number owned now	How often is it used in cattle farm? (1 = less than quarter of the time; 2 = between quarter and half of the time; 3 = between half and three quarter of the	When was it purchased or constructed? (month and year)	Initial purchase price or construction cost (US$)	Lifespan (years)	Insurance costs paid for the asset in the last 12 months (US$)	Taxes paid on the asset in the last 12 months (US$)

			time;<br>4 = more than three quarter of the time)					
Beef cattle fence								
Kraal								
Calf pen								
Store for farm inputs								
Dip sprayer								
Chaff cutter								
Wheel barrow								
Truck								
Pick-up								
Tractor								
Other (specify)								

Note: *If there are more than one type of any asset (e.g., two farm stores or dip sprayers), the enumerator should fill details of each asset on a separate row under the other category.*

SECTION G: Other Inputs and Services

(i) Land

17. What is the approximate size of your farm land (excluding homestead)? ______ acres

18. Which one of the following land tenure systems do you have on your farm? (Tick one option)
   a) Individual owned with title deed/allotment letter _____________
   b) Individual owned without title deed/allotment letter ___________
   c) Communal with title deed/allotment letter ___________________
   d) Communal without title deed/allotment letter ________________
   e) Mixed/other (specify, e.g., part individually-owned and partially communal)____________

(ii) Breed types and breeding method

19. What is the main cattle breed kept on your farm? (Tick one option)
   a) Local breed e.g ______
   b) Crossbreed ______________________
   c) Exotic e.g._________

20. Which cattle breeding method is normally used in the farm? (Tick one option)
   a) Natural breeding (controlled)____
   b) Natural breeding (uncontrolled)___
   c) Artificial insemination_________

(iii) Extension services

21) Did you get any livestock extension services in the last 12 months? ____ (1 = Yes, 2 = No), if NO, Go to Question 25.

22) Who was your main provider of livestock extension services in the last 12 months? (Tick one option)
   a) Government officer_______

b) Private provider e.g., Non-Government Organizations, private companies or individuals_________

23) How often does the main livestock extension service provider visit your farm? (Tick only one applicable option)

a) Weekly ____
b) Every two weeks ___
c) Once a month _____
d) Less than once a month ____

24) How often would you like the main extension service provider to visit your farm? (Tick one option)

a) Weekly _____________________
b) Every two weeks ______________
c) Once a month ________________
d) Less than once a month ________
e) Stop coming at all _____________

(iv) Veterinary advisory services

25) Did you receive any veterinary advisory services in the last 12 months? _____ (1 = Yes, 2 = No), if NO, GO to Question 27.

26) Where do you normally obtain veterinary advisory services from? (Tick one option)

a) Government officers______
b) Private providers e.g., Non Government Organizations, private companies or individuals_____

(v) Credit/loan

Cash loan

27) Did any household member try to get cash loan in the last 12 months? ______ (1 = Yes, 2 = No). If NO, GO to Question 31.

If YES, was the loan received? ____ (1 = Yes, 2 = No), if NO, GO to Question 35.

28) What were the sources of cash loan? (Tick all that apply).

a) Bank___
b) Cooperative society____
c) NGO) _____
d) Self help group_______
e) Family________
f) Neighbour______
g) Other (specify)____

29) Was the cash loan mainly used in (Tick one option):

a)Beef cattle enterprise? ________
b) Crop enterprise? _________
c) Other purposes, e.g., food, fees, medical bills? ______

30) Has the entire cash loan been repaid? ____ (1 = Yes, 2 = No)

In kind loan

31) Did any household member try to get loan in kind (e.g., machinery, equipment, feeds, veterinary drugs and livestock) in the last 12 months? (1=Yes, 2= No). If NO, GO to Question 38. If YES, was the loan received? ____ (1 = Yes, 2 = No), if NO, GO to Question 34.

32) Was the in kind loan mainly used in (Tick one option):

a) Beef cattle enterprise? ________
b) Crop enterprise? _________
c) Other purposes, e.g., food, fees, medical bills? ______

33) Has the entire in kind loan been repaid? ____ (1 = Yes, 2 = No)

SECTION H: Market Outlets

34) Which one of the following do you normally sell your cattle to? (Tick one option)
  a) Open market centre _____
  b) Slaughterhouses/butcheries ____
  c) Tajikistan Meat Commission (TMC) ____
  d) Middlemen/traders/brokers
  e) Private exporter e_____
  f) Other e.g., neighbour, breeder (specify) ____

35) What is the approximate distance from your farm to where you normally sell cattle? ________ Km

36) What is the type of road from your farm to where you normally sell beef cattle? (Tick one option)
  a) Tarmac_____
  b) Murram____
  c) Other, i.e., no tarmac or murram______

37) How would you describe the condition of the road from your farm to where you normally sell beef cattle? (Tick one option)
  a) Good, i.e. easily passable most of the time _________
  b) Poor, i.e., pot holed or muddy or rough most of the time _______

38) Do you normally sell cattle through prior arrangement (contract agreement)? ______ (1 = Yes, 2 = No), if NO, Go to Question 40.

39) Does the contract agreement include the following?
  a) Price ___ (1 = Yes, 2 = No)
  b) Transportation/delivery ___ (1 = Yes, 2 = No)
  c) Other (specify) _______________________

SECTION I: Market Information

40) Do you normally receive market information on beef cattle (e.g., on prices of beef cattle) before you go the market place? _____ (1 = Yes, 2 = No). If NO, GO to Section J.

41) How frequently do you normally receive the market information? (Tick one option)
  a) Daily ______
  b) Once a week ____
  c) Every two weeks ______
  d) Once a month _____
  e) Less than once a month _____

42) How important have the following channels been in enabling you to get market information during the last 12 months? (*Tick the relevant box for each source of information*)

**Source of information**	**Relative importance**			
	Not Applicable	*1 = Not Important*	*2 = Moderately Important*	*3 = Very Important*
Mobile phone				
Workshops/meetings				
Television				
Radio				
Internet				
Newspapers				
Advertisements/memos on notice boards				
Visiting friends and neighbours				

Others (specify)				

***Note**: Not Applicable means it was not used at all.*

SECTION J: Choice Experiment

43) Please indicate your opinion on the following statements, on a scale of 1 to 5 (where 1 = strongly disagree, 5 = strongly agree). *Tick one box for each statement*.

**Statement**	*1 = Strongly Disagree*	*2= Disagree*	*3 = Neither (undecided)*	*4= Agree*	*5 = Strongly Agree*
a) I consider cattle diseases as a serious problem to farming					
b) I am satisfied with current disease control programmes					

44) During previous severe outbreaks of cattle diseases and shortage of pasture, I mainly took the following action (*Tick one option*):

a) Sold beef cattle___________
b) Slaughtered beef cattle______
c) Moved cattle to safer areas with pastures__________
d) None of the above____________

45) Is pasture available and enough? ______ (1 = Yes, 2 = No).

THANK YOU FOR YOUR VALUABLE INFORMATION
