# Supplementary material for: Evaluating profitability of beef cattle farming and its determinants among smallholder beef cattle farmers in the Baljovan District of Khatlon region, Tajikistan
Source: PLoS One. 2022 Sep 13;17(9):e0274391. doi: 10.1371/journal.pone.0274391 (PMC9469962; doi:10.1371/journal.pone.0274391)
Supplement: Attachment — Submitted filename: PONE-D-21-37158_reviewer.pdf [file pone.0274391.s002.pdf]

PLOS ONE

Evaluating Profitability of Beef Cattle Farming and its Determinants among Smallholder Beef Cattle Farmers in the Baljovan District of Khatlon Region, Tajikistan

--Manuscript Draft--

**Manuscript Number:**	PONE-D-21-37158
**Article Type:**	Research Article
**Full Title:**	Evaluating Profitability of Beef Cattle Farming and its Determinants among Smallholder Beef Cattle Farmers in the Baljovan District of Khatlon Region, Tajikistan
**Short Title:**	Evaluating Profitability of Beef Cattle Farming and its Determinants among Smallholder Beef Cattle Farmers
**Corresponding Author:**	Farrukh Jobirov, PhD student<br>Jilin Agricultural University<br>Changchun, Jilin CHINA
**Keywords:**	Beef cattle; profit maximization; profitability, gross margin analysis; ordinary least squares (OLS) regression models; improved income; smallholder beef cattle farmers; poverty reduction; Tajikistan
**Abstract:**	In Tajikistan, owning beef cattle is an important survival mechanism for smallholder farmers for alleviating poverty. Therefore, beef cattle farming enterprises should indeed strive to maximize profit to excel and flourish in a free economy. Nevertheless, smallholder beef cattle farmers are notorious for inefficient profit maximization. Thus, this study was set to evaluate the profitability of beef cattle farming and its determinants to enhance profit maximization among smallholder beef cattle farmers in the Baljovan District of Khatlon region, Tajikistan. A total of 388 farming households were chosen at random and purposive for the study. The cross-sectional gathered information through questionnaires was analyzed by using descriptive, gross margin (GM), and ordinary least squares (OLS) regression models. Based on the descriptive analyses, the mean age of beef cattle farmers (farmers) was 52.73 years, with a household size of 7.07 members. The beef cattle farmers had an average of 18.23 cattle herd size with 8.54 years of farming experience. The average land area possessed by farmers was 10.59 hectares. Among farmers, men (98.2%) dominated beef cattle farming activities. Around 83.8% of farmers had a college grade (higher literacy). Besides that, around 89.4% of farmers had access to farm credits. However, only 71.4% of farmers used farm credit points to produce beef cattle. Most of the farmers (89.7%) had access to accurate market information; allowing 75.8% of farmers to sell their beef cattle to open market (profitable) outlets rather than middlemen/traders/brokers. About 89.4% had access to veterinary services. Additionally, about 82.7% of farmers acknowledged the availability of pasture for grazing, which motivated 87.6% of farmers to be involved in selling contracts. Furthermore, economic investigation results revealed that on average, farmers had a gross margin (GM-profit) of 353.77 US$ per cattle, with feed costs (58.6%) and medications costs (26.1%) accounting for the largest share of total variable costs. Meanwhile, profitability among farmers was positively and statistically significant at $P < 0.05$ influenced by education level, family size, farming experience, medications expenses, and access to credits. This study concluded that beef cattle production is a feasible business. However, the potential for increased profitability is significant if existing resources are efficiently coordinated and production expenses, notably feed and healthcare costs, are minimized. Thus, the government should develop additional measures for addressing concerns such as capacity building, suitable and freely available pasture as well as health management, to boost beef cattle profitability among farmers in Tajikistan.
**Order of Authors:**	Farrukh Jobirov, PhD student
	Zhang Yuejie, Professor
	Cornel Anyisile Kibona, PhD in Agricultural Economics and Management
**Additional Information:**	
**Question**	**Response**

Powered by Editorial Manager® and ProduXion Manager® from Aries Systems Corporation

**Financial Disclosure**<br><br>Enter a financial disclosure statement that describes the sources of funding for the work included in this submission. Review the submission guidelines for detailed requirements. View published research articles from *PLOS ONE* for specific examples.<br><br>This statement is required for submission and **will appear in the published article** if the submission is accepted. Please make sure it is accurate.<br><br>**Unfunded studies**<br>Enter: *The author(s) received no specific funding for this work.*<br><br>**Funded studies**<br>Enter a statement with the following details:<br>• Initials of the authors who received each award<br>• Grant numbers awarded to each author<br>• The full name of each funder<br>• URL of each funder website<br>• Did the sponsors or funders play any role in the study design, data collection and analysis, decision to publish, or preparation of the manuscript?<br>• **NO** - Include this sentence at the end of your statement: *The funders had no role in study design, data collection and analysis, decision to publish, or preparation of the manuscript.*<br>• **YES** - Specify the role(s) played.<br><br>* typeset	Yes;<br>This research is supported and funded by the National Beef Cattle Industrial Technology System and Industrial Economy Research Project under the Ministry of Agriculture in the People's Republic of China (PRC) (CARS-37).<br>The funders had no role in study design, data collection and analysis, decision to publish, or preparation of the manuscript.
**Competing Interests**<br><br>Use the instructions below to enter a competing interest statement for this submission. On behalf of all authors, disclose any competing interests that could be perceived to bias this work—acknowledging all financial support and any other relevant financial or non-financial competing interests.<br><br>This statement is required for submission	The authors have declared that no competing interests exist.

and **will appear in the published article** if the submission is accepted. Please make sure it is accurate and that any funding sources listed in your Funding Information later in the submission form are also declared in your Financial Disclosure statement.<br><br>View published research articles from *PLOS ONE* for specific examples.<br><br>**NO authors have competing interests**<br><br>Enter: *The authors have declared that no competing interests exist.*<br><br>**Authors with competing interests**<br><br>Enter competing interest details beginning with this statement:<br><br>*I have read the journal's policy and the authors of this manuscript have the following competing interests: [insert competing interests here]*<br><br>* typeset	
**Ethics Statement**<br><br>Enter an ethics statement for this submission. This statement is required if the study involved:<br><br>• Human participants<br>• Human specimens or tissue<br>• Vertebrate animals or cephalopods<br>• Vertebrate embryos or tissues<br>• Field research<br><br>Write "N/A" if the submission does not require an ethics statement.<br><br>General guidance is provided below. Consult the submission guidelines for detailed instructions. **Make sure that all information entered here is included in the Methods section of the manuscript.**	All ethical issues were addressed appropriately during the information-gathering procedures. The Jilin Agricultural University Graduate Research Ethics Committee in China originally authorized this research. The Ministry of Agriculture then authorized it for the order id (RP.2021/89/25). Thereafter, initially, the respondents' consent was acquired orally, and then the accurate information in the consent letter was conveyed to all targeted respondents. Before their involvement in the case, the respondents were invited to fill out and execute the documents as verification of their agreement to engage in the survey. The consent was then authorized by the Zone Executive Officer (ZEO). All respondents were assured that they had the option to withdraw from the event at any stage.

Powered by Editorial Manager® and ProduXion Manager® from Aries Systems Corporation

**Format for specific study types**<br><br>**Human Subject Research (involving human participants and/or tissue)**<br>• Give the name of the institutional review board or ethics committee that approved the study<br>• Include the approval number and/or a statement indicating approval of this research<br>• Indicate the form of consent obtained (written/oral) or the reason that consent was not obtained (e.g. the data were analyzed anonymously)<br><br>**Animal Research (involving vertebrate animals, embryos or tissues)**<br>• Provide the name of the Institutional Animal Care and Use Committee (IACUC) or other relevant ethics board that reviewed the study protocol, and indicate whether they approved this research or granted a formal waiver of ethical approval<br>• Include an approval number if one was obtained<br>• If the study involved *non-human primates*, add *additional details* about animal welfare and steps taken to ameliorate suffering<br>• If anesthesia, euthanasia, or any kind of animal sacrifice is part of the study, include briefly which substances and/or methods were applied<br><br>**Field Research**<br><br>Include the following details if this study involves the collection of plant, animal, or other materials from a natural setting:<br>• Field permit number<br>• Name of the institution or relevant body that granted permission	
**Data Availability**<br><br>Authors are required to make all data underlying the findings described fully available, without restriction, and from the time of publication. PLOS allows rare exceptions to address legal and ethical concerns. See the PLOS Data Policy and FAQ for detailed information.	Yes - all data are fully available without restriction

Powered by Editorial Manager® and ProduXion Manager® from Aries Systems Corporation

A Data Availability Statement describing where the data can be found is required at submission. Your answers to this question constitute the Data Availability Statement and **will be published in the article**, if accepted.<br><br>**Important:** Stating 'data available on request from the author' is not sufficient. If your data are only available upon request, select 'No' for the first question and explain your exceptional situation in the text box.<br><br>Do the authors confirm that all data underlying the findings described in their manuscript are fully available without restriction?	
**Describe where the data may be found in full sentences. If you are copying our sample text, replace any instances of XXX with the appropriate details.**<br><br>• If the data are **held or will be held in a public repository**, include URLs, accession numbers or DOIs. If this information will only be available after acceptance, indicate this by ticking the box below. For example: *All XXX files are available from the XXX database (accession number(s) XXX, XXX.)*.<br>• If the data are all contained **within the manuscript and/or Supporting Information files**, enter the following: *All relevant data are within the manuscript and its Supporting Information files.*<br>• If neither of these applies but you are able to provide **details of access elsewhere**, with or without limitations, please do so. For example:<br><br>*Data cannot be shared publicly because of [XXX]. Data are available from the XXX Institutional Data Access / Ethics Committee (contact via XXX) for researchers who meet the criteria for access to confidential data.*<br><br>*The data underlying the results presented in the study are available from (include the name of the third party*	All relevant data are within the manuscript and its Supporting Information files.

and contact information or URL).<br>• This text is appropriate if the data are owned by a third party and authors do not have permission to share the data.<br><br>* typeset	
Additional data availability information:	

Evaluating Profitability of Beef Cattle Farming and its Determinants among Smallholder Beef Cattle Farmers in the Baljovan District of Khatlon Region, Tajikistan

Farrukh Jobirov[1,*], Zhang Yuejie[1], Cornel Anyisile Kibona[1, 2]

1 College of Economics and Management, Jilin Agricultural University, Changchun, Jilin, China,

2 Department of Agricultural Economics and Finance, Mwalimu Julius. K. Nyerere University of Agriculture and Technology, Musoma, Tanzania

*Corresponding Author: jobirovfarrukh@gmail.com (FJ)

Abstract

In Tajikistan, owning beef cattle is an important survival mechanism for smallholder farmers for alleviating poverty. Therefore, beef cattle farming enterprises should indeed strive to maximize profit to excel and flourish in a free economy. Nevertheless, smallholder beef cattle farmers are notorious for inefficient profit maximization. Thus, this study was set to evaluate the profitability of beef cattle farming and its determinants to enhance profit maximization among smallholder beef cattle farmers in the Baljovan District of Khatlon region, Tajikistan. A total of 388 farming households were chosen at random and purposive for the study. The cross-sectional gathered information through questionnaires was analyzed by using descriptive, gross margin (GM), and ordinary least squares (OLS) regression models. Based on the descriptive analyses, the mean age of beef cattle farmers (farmers) was 52.73 years, with a household size of 7.07 members. The beef cattle farmers had an average of 18.23 cattle herd size with 8.54 years of farming experience. The average land area possessed by farmers was 10.59 hectares. Among farmers, men (98.2%) dominated beef cattle farming activities. Around 83.8% of farmers had a college grade (higher

literacy). Besides that, around 89.4% of farmers had access to farm credits. However, only 71.4% of farmers used farm credit points to produce beef cattle. Most of the farmers (89.7%) had access to accurate market information; allowing 75.8% of farmers to sell their beef cattle to open market (profitable) outlets rather than middlemen/traders/brokers. About 89.4% had access to veterinary services. Additionally, about 82.7% of farmers acknowledged the availability of pasture for grazing, which motivated 87.6% of farmers to be involved in selling contracts. Furthermore, economic investigation results revealed that on average, farmers had a gross margin (GM-profit) of 353.77 US$ per cattle, with feed costs (58.6%) and medications costs (26.1%) accounting for the largest share of total variable costs. Meanwhile, profitability among farmers was positively and statistically significant at $P < 0.05$ influenced by education level, family size, farming experience, medications expenses, and access to credits. This study concluded that beef cattle production is a feasible business. However, the potential for increased profitability is significant if existing resources are efficiently coordinated and production expenses, notably feed and healthcare costs, are minimized. Thus, the government should develop additional measures for addressing concerns such as capacity building, suitable and freely available pasture as well as health management, to boost beef cattle profitability among farmers in Tajikistan.

Keywords: Beef cattle; profit maximization; profitability, gross margin analysis; ordinary least squares (OLS) regression models; improved income; smallholder beef cattle farmers; poverty reduction; Tajikistan.

1. Introduction

In Tajikistan, owning beef cattle is an important survival mechanism for smallholder farmers [1, 2]. Beef cattle are kept by families for several purposes, which vary based on their

prosperity, environment, and endowment. Beef cattle give numerous advantages to millions of agricultural households in underdeveloped countries, from monetary revenue to food, from dung to draft power in farming, benefiting people's lives via multiple channels [1]. Beef cattle, in particular, can contribute to prosperity by generating income and in-kind revenue from the sale of cattle and/or the marketing and utilization of mammal foodstuffs including meat and milk [1]. Furthermore, cattle serve as a safety net in the form of liquid assets, and it is commonly regarded also as a form of investment and security attributed to the fact that the sale of cattle offers immediate cash influx to cope with unanticipated financial instability [3, 4]. Additionally, owning cattle can facilitate access to legal and tacit loans since they can be used as collateral [5, 6]

Tajikistan's turbulent history has had a negative influence on the agriculture sector's current situation [[illegible] Its passage from the Soviet Union to independence had been an arduous one, hampered by a five-year civil conflict that damaged infrastructures and interrupted marketplace links. Deforestation and overgrazing have worsened soil quality and raised the danger (threats) of soil degradation, rock slides, and floods [2]. The threats have arisen as a result of low infrastructure improvements, a lack of suitable laws and policy frameworks for grazing and forest management, and restricted institutional support. ~~This uncertainty has a significant influence~~ on the beef cattle industry [2]. ~~Furthermore,~~ according to a study published by the Republic of Tajikistan [2], beef cattle stocks have increased to levels greater than in the immediate pre-independence period, and beef cattle husbandry is an operation in which practically almost every poor population participates. Because of the increase in stocks accompanied by the decrease in feed availability, feed per beef cattle has declined considerably, as has beef cattle performance. There are numerous barriers to the advancement of the beef cattle

industry, including a lack of human resources, underprivileged pasture governance, poor planning of community beef cattle, an absence of feed during the cold season, environmental devastation, and limited access to healthy forages grains and infrastructural facilities, all of which are exacerbated by climate change [2].

Investing in initiatives to address the challenges to improved beef cattle efficiencies will have an important influence on smallholders' dietary diversity, food security status, revenue, jobs, and livelihood opportunities [7, 8]. Due to the general intense relationship between beef cattle efficiency and relevant use of pastures, a concomitant focus on grazing strategic planning components of beef cattle production is required [8]. The accessibility of wintertime fodder plus early summer pastures, which do not fulfill requirements, are the production constraining variables. Summertime grasses, on the other hand, supplied substantial grazing excess compared to present requirements. As a result, there has been a periodic imbalance between supply and demand [7, 8]. Steadily increasing beef cattle herds in certain areas have put further strain on improperly organized communal grazing. Because the growth period for calves after weaning frequently relies on this grassland and grazing on other peripheral sites, this adds to feeder calves' poor health and productivity [9]. Despite increasing beef cattle populations, relative low ownership implies that beef cattle production is still mostly a subsistence operation [9].

Tajikistan produces the majority of its meat from bulls born to mixed-breed cattle with a focus on dairy qualities. There are various particular beef breeds in the nation; however, the majority of beef cattle are native breeds (local breeds) mixed with dual-purpose breeds like Brown Swiss or Brown Carpathian [10].

Recognizing the need to re-establish grassland and minimize deterioration, thus further improving acreage for pastures in the long run for the financial stability of beef cattle production

~~in Tajikistan, the Tajik government imposed the Livestock and Pasture Development Project I & II (LPDP I & II), which was supported by the International Fund for Agricultural Development (IFAD), from 2015 to 2017 [2],~~ The initiative addressed around 38,000 farmers in at least 200 settlements throughout five districts of the Khatlon region, all of which have significant poverty rates and the opportunity for improved beef cattle productivity. The project's key target respondents included: (i) smallholder livestock communities; (ii) commercial veterinarian service providers and micro enterprises with the ability to assist rural families and local farmers; and (iii) women-headed families and impoverished women. The project's overall purpose was ~~to help to~~ alleviate poverty in Khatlon Region. Moreover, the explicit purpose was to improve the nutritional status and income of about 38,000 impoverished families by increasing beef cattle output [2].

~~Furthermore,~~ according to the Republic of Tajikistan's ~~[2]~~ press release, the ~~project's~~ investments and operations were carried out ~~through three aspects;~~ (i) institutional advancement: the primary goal of this aspect was to build the strength of the focused public sector and civic groups to be more reliable and successful in developing pro-poor grassland monitoring systems; (ii) performance improvement and optimized animal health: the main goal of this aspect was to improve access to veterinary care, resulting in lower morbidity and enhanced performance of herds due to lower incidence rates of diseases; (iii) grassland advancement and expansion for disaster mitigation: the primary goal with this aspect was to improve access to more proficient and climate-resilient grassland zones, as well as diverse revenue potentials for beef cattle societies, through self-sustaining, community-led biodiversity conservation. ~~The major anticipated impact was improved living standards, resulting in alleviating poverty among~~ beef cattle farmers in selected districts of the Khatlon region and the nation as a whole.

Despite efforts to increase the economic viability of Tajikistan's beef cattle industry and thereby eradicate poverty, Tajikistan remains a food-insecure state, having 46% of Tajiks living in extreme poverty [11]. Poverty is especially severe in rural parts, where the people are mostly reliant on farming, livestock, and remittances for a living and food supply [11]. This demonstrates the poor economic gains, effectiveness, and modernization level of beef cattle production in Tajikistan, notably in the Khatlon region, where the project was carried out. Additionally, despite 27 years of solid economic progress, Tajikistan is ~~nevertheless~~ the poorest and least industrialized nation in Central Asia. The economy is still in its early ~~stages~~, with a low value-added (including productivity improvement to beef cattle) and a limited export foundation [11].

Profit maximization is one of the primary goals of every business company (beef cattle farming enterprise) for the long performance and sustainability [12, 13]. Profitability is the act of profit-earning abilities, which is a significant aspect of a company's survival (beef cattle farming business) [12, 13]. Furthermore, profit has a substantial impact on the company's achievement of other business future such as economic and social development, technology, jobs, and technical advancement [12, 13].

~~Enterprises~~ (beef cattle farming enterprises) should indeed strive to maximize profit to excel and flourish in a free economy, or they will be forced into bankruptcy for failing to create adequate money [14]. ~~Enterprises~~ (beef cattle farming enterprises) are having difficulty achieving the requisite profit due to increased competitive pressures and inefficiency. As a result, the issue of what variables influence profitability should be a top concern for academicians and researchers, comprising decision-makers, investment companies, debtors, and policymakers dealing with beef cattle farming businesses [13].

The study of the factors of profitability has increased in popularity throughout time in a variety of research fields. Experts of business strategy, economics, and accounting, believe that a company's internal resources have a substantial influence on its profitability [12, 15, 16]. Generally, profitability (performance) is an important measure of the effectiveness of any dividend enterprise (profit-oriented beef cattle farming enterprise). The enterprise (beef cattle farming enterprise) goal (profitability) is achieved as an outcome of the effective utilization of the set of resources (determinants) for the aim of maximizing income to the greatest extent feasible.

There are almost no recorded studies that have examined the profitability of Tajikistan's beef cattle farming enterprise among smallholder farmers. Therefore, this study was set to assess the economic benefits of beef cattle farming and its determinants among beef cattle farmers in the Baljovan District of Khatlon region, Tajikistan, specifically to (i) analyze the socioeconomic characteristics of beef cattle farmers and (ii) examine the profitability of beef cattle farming and its determinants among smallholder beef cattle farmers. Thus, the purpose of this study was to convey an awareness of how beef cattle farming (business) performs and what it contributes to the economy of the smallholder beef cattle farmer in Tajikistan for poverty reduction.

2. Materials and Methods

2.1. Description of the Study Area

The study was conducted in the Baljovan District of the Khatlon Region. The region and its district were selected ~~as a study~~ because Tajikistan implemented Livestock and Pasture Development Project I & II (LPDP I &II). Khatlon region, particularly the selected district has a potential for increased beef cattle productivity, with a high poverty level. The Khatlon region is

located in the southwest of the country, with a population of 2.64 million, or approximately one-third of the country's total population.

2.2. Ethical Considerations

All ethical issues were addressed appropriately during the information-gathering procedures. The Jilin Agricultural University Graduate Research Ethics Committee in China originally authorized this research. The Ministry of Agriculture then authorized it for the order id (RP.2021/89/25). Thereafter, initially, the respondents' consent was acquired orally, and then the accurate information in the consent letter was conveyed to all targeted respondents. Before their involvement in the case, the respondents were invited to fill out and execute the documents as verification of their agreement to engage in the survey. The consent was then authorized by the Zone Executive Officer (ZEO). All respondents were assured that they had the option to withdraw from the event at any stage.

2.3. Sampling Procedures and Sample Size

Purposive and randomized sampling was used to select participants at different phases, with a sample size of 388 beef cattle farmers generated by a multistage stage sampling technique. In the first stage, the Khatlon Region zone was purposively selected from five major beef cattle-producing regions in Tajikistan. In the second stage, Baljovan District was purposively selected from five districts of Khatlon Region covered by Livestock and Pasture Development Project I & II (LPDP I &II). The selected district has great potential beef cattle-producing areas. In the third stage, five villages (strata) (Baljyvon, Tojikiston, Sayf Rahim, Safar Amirshoev, and Sari Hosor), were randomly selected. Finally, the number of respondents (sample size) selected from each village (stratum) was determined by utilizing the percentage proportion (see Table 1). This study

applied Slovin's formula [17] to the targeted (N = 12,761) beef cattle farmers to determine a randomly selected sample size of 388 representative beef cattle farmers. The Slovin's formula [17] as applied by Kibona and Yuejie [18] is mathematically expressed as;

$$n = \frac{N}{1 + Ne^2} = \frac{12{,}761}{1 + 12{,}761\ (0.05)^2} = 387.81\ \approx 388 \qquad (1)$$

Whereby *N* is the targeted population size, *n* is a sample size, and *e* is the error tolerance level or is the level of precision provided by Yamane [19] to determine the required sample size at 95% confidence level and 90% level of precision.

Table 1. Distribution of the sample size.

District	Villages	Population	Percentage Proportion	Sample
Baljovan	Baljyvon	2,571	20.15	78
	Tojikiston	2,540	19.90	77
	Sayf Rahim	2,555	20.02	78
	SafarAmirshoev	2,560	20.06	78
	Sari Hosor	2,535	19.87	77
**Total**		**12,761**	**100**	**388**

2.4. Techniques for Data Collection

The structured questionnaire and interview approaches were used to gather cross-sectional primary data from smallholder beef cattle farmers. The structured questionnaires and interviews collected data on (i) socioeconomic characteristics of smallholder beef cattle farmers; (ii) expenses of beef cattle production (variable costs) and revenue; (iii) beef cattle ownership status (including breed types and breeding methods); (iv) sales of beef cattle (marketing); distinctively, the market outlets (the distribution channels), the number of beef cattle sold, the selling price, and the contract selling; and (v) availability of pasture and coping strategies during severe outbreaks of cattle diseases and shortage of pasture.

2.5. Data Analysis

Both descriptive statistical analysis and econometric models were used in this study. The information gathered through questionnaires and interviews was coded and analyzed using Excel and SPSS v. 22.

2.5.1. Descriptive Data Analysis

Descriptive statistical analysis involving means, maximum, minimum, standard deviations, frequencies, and percentages were used to examine the: (i) socioeconomic characteristics; (ii) beef cattle ownership status; (iii) market outlets (the distribution channels); (iv) selling price; (v) contract selling; and (vi) availability of pasture.

2.5.2. Economic Data Analysis Models

The economic data were analyzed using a gross margin analysis (GMA) and ordinary least squares (OLS) multiple linear regression models as described below;

2.5.2.1. The Gross Margin Analysis ~~(GMA)~~ model

The gross margin analysis ~~(GMA)~~ model was used to examine the profitability of beef cattle farming among smallholder beef cattle farmers. The gross margin (GM) was computed by subtracting the total revenue (TR) from the total variable cost of beef cattle farming (TVC). This formula is formally described as follows:

$$GM_i = TR_i - TVC_i \quad (2)$$

Where, TR_i stands for the total revenue of farming per beef cattle in US $, TVC_i is the total variable cost per beef cattle in US $, and GM_i represents the gross margin of farming per beef cattle in US$. In addition, according to Kibona and Yuejie, [20], to discover profit value, it is necessary to first determine physical values and unit price for input and output variables,

followed by a computation of producing expenses and revenues. As a result, the functional equation for the gross margin analysis (GMA) model is described below.

$$\mathrm{GM} = \sum_{i=1}^{n} Y_i P y_i - \sum_{j=1}^{m} X_j P x_j \tag{3}$$

Here; *GM* stands for the gross margin per beef cattle, $\sum_{i=1}^{n} Y_i P y_i$ is the total revenue (TR) of *n* beef cattle, $P y_i$ is the beef cattle market price, Y_i is the total number of beef cattle sold, $\sum_{j=1}^{m} X_j P x_j$ is the total cost of *m* variable inputs per beef cattle, X_j is the amount of the jth variable input (j = 1, 2, 3…*n*, *m* inputs), $P x_j$ is the cost of inputs utilized per unit/price per unit of a variable input, and $\sum$ is the summation symbol.

Additionally, the total variable operating costs (TVCs) were calculated by adding the expenses of husbandry labor (excluding family labor costs), medicines (treatment), feeds (silage, fodder, and other feeds like soybean meal and urea), mineral salts and vitamins, spraying or dipping, veterinary services, and some other expenses for sales and logistics. Furthermore, for this study, fixed costs were not included in gross margin estimates since fixed costs are non-quantifiable owing to the traditional managerial style utilized by smallholder beef cattle farmers [20].

2.5.2.2. The Ordinary Least Squares (OLS) Multiple Linear Regression Model

The Ordinary Least Squares (OLS) model was used to analyze the determinants of profitability of the beef cattle farming business among smallholder beef cattle farmers. The model is precisely specified as follows:

$$Y_i = \beta_0 + \beta_1 X_1 + \beta_2 X_2 + \beta_3 X_3 + \ldots\ldots\ldots.. + \beta_n X_n + \varepsilon_i \tag{4}$$

Where

Yi = gross margin-GM (profit) of ith smallholder beef cattle farmer per beef cattle (US$)

β_0 = constant

$\beta_1,\ldots,.. \beta_n$ = coefficients to be estimated

$X_1,\ldots, X_n$ = independent variables

ε_i = error term, this represents all factors that influence the variance but are not depicted by the explanatory variables [21].

As cited by Kibona and Yuejie, [18], the ordinary least squares (OLS) approach is a numerical modeling tool that is used to explain the relationship between a continuous dependent variable (gross margin- profit) and several independent factors (determinants of profitability) [22]. The multiple linear regression approach was used to determine the intensity and significance of the relationship among profitability per beef cattle and factors that are anticipated to affect profitability [21]. These include the variable costs (feed costs, husbandry labor costs, medicines (treatment) costs, and sales and logistics costs), education level of a farmer, family size, cattle herd size, land owned, experience in farming, access to veterinary services, access to credits, pasture availability, selling contract, marketing channels, and access to market information.

Therefore, the abbreviated OLS regression model for the determinants of profitability of beef cattle farming industry among smallholder beef cattle farmers were further described as follows:

$$
\begin{aligned}
&Gross\ margin\ (profit) \\
&\quad = \beta_0 + \beta_1 Feed\ costs + \beta_2 Husbandry\ labour\ costs + \beta_3\ Medicines\ costs \\
&\quad + \beta_4\ Sales\ and\ logistic\ costs + \beta_5 Education\ level + \beta_6 Familiy\ size \\
&\quad + \beta_7 Cattle\ herd\ size + \beta_8 Land\ owned + \beta_9 Experience\ in\ farming \\
&\quad + \beta_{10} Access\ to\ veterinary\ services + \beta_{11} Access\ to\ credits \\
&\quad + \beta_{12} Pasture\ availability + \beta_{13} Selling\ contract + \beta_{14} Marketing\ channels \\
&\quad + \beta_{15} Acces\ to\ market\ information \\
&\quad + \varepsilon \qquad (5)
\end{aligned}
$$

According to Mlote et al., [21], a failure of the assumptions underlying OLS regression analysis may threaten the validity of the regression results. Any failure of the hypotheses (explanatory variables not having a normal distribution, autocorrelation, heteroscedasticity, and multicollinearity) renders the component estimations invalid for interpretation. Therefore, the model was verified for validity using SPSS v. 22 statistical analyses. The Variance Inflation Factor (VIF) resulted in a score of 1.31, confirming the absence of multicollinearity between the dependent and independent variables [23].

2.6. Theoretical Framework

The theory of Resources Based Views (RBV) provided the framework for this study. According to Barney [12] and Wernerfelt [24] and ~~Resources Based Views~~ (RBV) theory is a concept that considers resources to be critical to the better company (better beef cattle farming enterprise) performance (profitability). The resources allow the business (beef cattle farming business) to achieve and maintain a competitive edge. To be useful, resources ought to be beneficial, unique, and pricey to imitate (costly), as well as better controlled.

Beneficial: As per Wernerfelt [24], resources are beneficial if they assist firms (beef cattle farming business) in maximizing the price provided to consumers. This is accomplished via enhancing distinctiveness and/or lowering input costs (minimizing the cost of farming).

Resources that are unable to achieve this need are at a competitive disadvantage. Unique: As per Curran et al., [25], and Wernerfelt [24], unique resources are those that can be obtained on an exceptional basis by one or a few enterprises (beef cattle farming enterprises). Competitive balance occurs when many firms (beef cattle farming enterprises) have the same resources or competence [12, 24]. Costly: If an enterprise (beef cattle farming enterprise) wishes to maintain a competitive edge, the resource ought to be costly to replicate or substitute for a competitor. Therefore, resources alone do not provide a competitive edge to an enterprise (beef cattle farming enterprise) if they are not controlled to maximize their worth. Only the enterprise that can utilize beneficial, unique, and costly to imitate resources will be able to maintain a competitive edge in the long run [24, 26].

The Resources Based View (RBV) analyzes company performance in different ways, such as explaining profitability primarily based on distinct firm-level attributes, resources, and competencies [24, 27]. As per the Recourses Based View concept, enterprises take divergent historical pathways, resulting in diverse competency that impacts their capacities in various ways [24, 27]. Progressive companies (beef cattle farming enterprises) in a sector are effective since they have access to a variety of resources and use them efficiently to obtain a competitive edge. Along with this perspective, resources include all internal and external resources such as brand names (beef cattle farming method), strengths, credits, stock (herds size), cash (capital-variable costs), skilled people (education level), location (distance to the market), an appropriate management system, entity good products, information (access to market information), and skills, and even cost-effective strategies that are moderately connected to the enterprise (beef cattle farming enterprise) [24]. As a result, the functional purpose of each enterprise (beef cattle farming enterprise) is to form its distinctive mix of resources to strengthen its economic

capability, resulting in increased profit (profit maximization) [12, 28]. The concept is relevant in this research because it demonstrates how an enterprise uses its internal resources, as well as its capacity to interact with the surroundings, to generate greater profit. In addition, internal resources such as wealth, skilled workforce, and expert knowledge, as well as the ability to access the market, credits, and market information through trading with the treasury, socializing with customers on social media, and other abilities such as company size, age of the firm, and firm site, are the primary resources that are linked with the determinants of profitability. In this study, determinants of profitability were investigated using resources-based theory. As a result, there is a strong relationship between theory and determinants of profitability.

3. Results and Discussions

3.1. Descriptive ~~Investigation~~ Results

3.1.1. The Socioeconomic Characteristics of Smallholder Beef Cattle Farmers

Table 2 results reveal that smallholder beef cattle farmers had an average age of 52.73 years. This suggests that farmers were of working age, which is vital in the successful implementation of intensive beef cattle production practices for more profit [18]. Farmers had an average family size of 7.07 members. This shows that farmers have a greater labor force opportunity for beef cattle production and sales operations, thereby increasing the profitability of the beef cattle farming business [18]. Moreover, the farmers had an average of 18.23 cattle herd size with 8.54 years of farming experience. It signifies that beef cattle production is pretty new between most farming households, necessitating the development of knowledge and expertise for increased efficiency. The findings of this study further indicated that the average land area possessed by beef cattle farmers was 10.59 hectares.

Furthermore, results showed that men dominated beef cattle farming activities among smallholder beef cattle farmers. There were around 98.2% males and 1.8% females among the 388 sampled respondents. To eliminate gender inequalities, females should indeed be motivated to participate in beef cattle production because practically all females are active in production activities, while males relocate from rural to urban areas in search of work [20, 29]. The findings also found that the majority of farmers had a high degree of literacy. Around 83.8% of farmers had a college degree, while 7.0 % and 9.3% had elementary and secondary education, respectively. Besides that, this study indicates that around 89.4% of farmers had access to farm credits, whereas 10.6% did not. Despite this high level of access to farm credits, only 71.4% of farmers used farm credit points to produce beef cattle. Regarding the accessibility to veterinary services, the statistics reveal that 89.4% of farmers had access. This means that there is a high level of accessibility to veterinary care. This increase in access to veterinary services is critical for lowering the cost of obtaining consultations required for increased beef cattle production [20].

According to the findings, 89.7% of farmers had access to market information, while only 10.3 of farmers did not. This might imply that farmers were more accessible to accurate market knowledge, allowing 75.8% of farmers to sell their beef cattle to open market outlets (profitable outlets) rather than middlemen/traders/brokers. Farmers' revenues are eroded when beef cattle are sold to middlemen/traders/brokers. Accurate market information is critical for farmers looking to improve the performance (profitability) of their beef cattle business [18]. Additionally, about 82.7% of farmers acknowledged the availability of pasture for grazing, which encouraged 87.6% of farmers to get into a selling contract agreement. This helps to protect the interest of beef cattle. The abundance of grazing pasture is significant in beef cattle production, which increases the supply of beef cattle for sale [18].

Table 2. Socioeconomic characteristics of smallholder beef cattle farmers.

Continuous Parameters (N = 388)

**Parameters**	**Mean**	**Maximum**	**Minimum**	**Std.Deviation**
Farmer's age	52.73	70.00	33.00	9.37
Family size	7.07	12.00	3.00	2.07
Cattle herd size (local breed)	18.23	50.00	2.00	12.00
Experience in farming	8.54	19.00	2.00	4.64
Land owned (ha)	10.59	230.67	0.41	30.83

Categorical Parameters (N = 388)

**Parameters**	**Frequency**	**Percentage (%)**
**Gender**		
Male	381	98.2
Female	7	1.8
**Education level**		
Primary Educ.	27	7.0
Secondary Educ.	36	9.3
College Educ.	325	83.8
**Access to farm credits**		
Yes	347 (277)*	89.4 (71.4)*
No	41	10.6
**Access to market information**		
Yes	348	89.7
No	40	10.3
**Access to veterinary services**		
Yes	347	89.4
No	41	10.6
**Pasture availability and sufficient**		
Yes	321	82.7
No	67	17.3
**Marketing channels (Market outlets)**		
Open markets	294	75.8
Slaughterhouses/butcheries	73	18.8
Tajikistan Meat Commission	7	1.8
Middlemen/traders/ brokers	14	3.6
**Selling contract**		
Yes	340	87.6
No	48	12.4

* *The figures in parentheses solely represent the frequency and percentage of farmers who utilized credits for cattle production*

3.2. Economic ~~Investigation~~ Results

3.2.1. The ~~Profitability~~ (gross margin) of Beef Cattle Farming among Smallholder ~~Beef Cattle~~ Farmers

~~Profitability (profit)~~ is the primary aim of every agricultural business, therefore profit is determined by calculating the gross margin (GM), and it measures the operating efficiency [30]. According to the findings in Table 3, farmers had a ~~gross margin~~ (GM) of 353.77 US$ per beef cattle on average. This indicates that beef cattle production is a viable (profitable) enterprise in the study area. This is in line with the observations of Kibona and Yuejie [20], Mafimisebi et al. [31], Nasiru et al. [32], and Okoruwa et al. [33], who found livestock farming to be a profitable venture.

~~Furthermore, according to~~ the findings, feed costs had the greatest operational expenditures, accounting for around 58.6% of the total variable production costs. It was followed by medications (treatment expenses) (26.1%), and husbandry labor costs (13.1%). Other expenditures were just under 2%, including vitamin and minerals salt costs (0.7%) and sales and logistics costs (1.3%). This suggests that the Livestock and Pasture Development Project I & II (LPDP I & II) undertaken in the research region had little impact on feed cost reduction; however, the potential to increase beef cattle farming profitability is great once feed costs are minimized. This implies that a decrease in production costs as a result of pasture availability (free or cheap available) would result in high profitability [20]. In addition, the significant cost of treatment or pharmaceuticals is a result of the incidence of animal illnesses in the research area. According to kibona and Yuejie [20], treating beef cattle following an outbreak of the illness is expensive. As a result, developing beef cattle-specific healthcare initiatives to address possible health issues is the greatest and only method to minimize cost; consequently increasing profitability [20].

Table 3. The gross margin (profitability) of beef cattle farming among smallholder beef cattle farmers (N = 388).

Parameters	The estimated value per cattle (US$) (N=388)			
**Cattle production variable costs**	**Mean**	**Max.**	**Min.**	**Std.Deviation**
Husbandry labor*	9.95(13.1)[1]	119.90	0.00	21.04
Medicines (treatment)*	19.85(26.1)	111.90	0.00	21.24
Feeds (silage, fodder, soybean meal, & urea)*	44.51(58.6)	383.70	0.00	74.66
Vitamins and mineral salts*	0.51(0.7)	6.40	0.00	1.51
Sales and logistics	1.11(1.5)	6.20	0.00	1.90
**Total variable cost (TVC)***	**75.92**	**511.50**	**1.80**	**103.36**
**Earn revenue and sales value**				
Selling price for cattle *	429.69	680.00	50.00	138.58
**Total revenue (TR) per cattle***	**429.69**	**680.00**	**50.00**	**138.58**
**Gross margin(GM) = (Total revenue - Total cost)***	**353.77**	**634.30**	**-356.60**	**231.75**

**At the time of sale, beef cattle on average were 4.5 years old. [1]The number in parentheses is the ratio of cost-share to total variable costs.*

3.2.2. The Factors Influencing the Profitability (gross margin) of Beef Cattle Farming among Smallholder Beef Cattle Farmers

Table 4 presents the results of a multiple regression analysis utilizing ordinary least squares (OLS) on the key parameters (variables) that determined the profitability of beef cattle farming among smallholder beef cattle farmers. The model was calculated utilizing SPSS v.22, and it fitted well and was statistically significant at $P < 0.05$. The model's adjusted R - squared = 0.833, signifying that the independent variables explain 83.3% of the variance in profitability per beef cattle among beef cattle farmers.

Holding other factors constant, variable costs (feed cost and sales and logistics costs) had a negative and statistically significant impact on profitability. A negative correlation reveals that when feed costs, as well as sales and logistics costs, rise, profitability per cattle falls by 0.713 U$ and 0.148 US$, respectively. Moreover, the negative values for sales and logistics indicated that a rise in these parameters would result in a fall in interviewees' profits, which might be attributable to the expensive cost of transportation in the research location. This observation is consistent with those of Nasiru [34] and Suleiman et al [35]. Furthermore, feed expenses, as well as sales and logistics costs, highlight the capital involved in the production and trading

operations. Marketing expenditures, on the other hand, indicate apparent transaction fees that may impede profit maximization [36].

Surprisingly, the coefficients for medications (treatment costs) had a positive influence on the profitability of beef cattle farming and were statistically significant at $P < 0.05$. A positive relation for medications costs indicates that cash invested in pharmaceuticals enhances beef cattle health (free of diseases and weight gain), which promotes beef cattle productivity and, as a result, beef cattle profitability.

The educational coefficient is positively significant. This validates an evident finding that almost educated farmers are quite cost-effective, and is also influential on the socio-cultural capital impacts that literacy may assist to organize. Education improves the ability to apply knowledge and the usage of ideas essential for cattle production [37]. The variable is also a predictor of the acceptance of advancements and new technologies required to boost farm output [36].

The profitability of beef cattle production was positively significantly related to family size. Huge families have the opportunities for much more family labor involved in production and sales operations, thereby boosting profitability. The number of people responsible for the day-to-day care of cattle is measured by labor. Farmers that have much personnel to care for the cattle can manage higher herd sizes, which increases the chances of improving profitability [36].

Given that grazing area, abundance is significant in beef cattle productivity and profitability, which improves the economic opportunities and effectiveness of beef cattle production [18]. This study, however, found that land ownership had a negative impact on the profitability of beef cattle production, and it was statistically significant at the 5% significance level. This suggests

that when ownership rises in rural regions, beef cattle production efficiency decreases because farmers opt to move land for crop cultivation rather than beef cattle farming.

Beef cattle herd size had a negative influence on beef cattle production profit yet was statistically significant at a 5% level of significance. The findings show that as herd size grows the profitability of beef cattle declines; it is difficult and expensive to manage larger herd sizes effectively. This finding is in line with koknaroglu et al., [38], who reported that fewer beef cattle per farm result in higher profitability per animal. Therefore, farmers should be motivated to minimize massive cattle herds, stay in grazing areas, and efficiently feed beef cattle before selling (production efficiency) for a higher return [18]. A profit-maximizing firm in agricultural production can produce and distribute superior products in the target marketplace at a financial benefit for the enterprise's existence [39].

The coefficient for farming experience was significantly positive at the 5% level. This indicates that farmers who have been raising beef cattle for a long time generated higher money as a result of their understanding of trends in beef cattle production and marketing. This result confirms Afolabi's previous results [40]. Farming experience highlights the power of social media platforms and linkages reinforced to improve the exploration of new clients [41]. Furthermore, farming experience provides information and producing practices that are recognized to be effective in production and marketing operations [42].

Exposure to farm credits had a positive impact on the profitability of beef cattle production, and it was statistically significant at a 5% level. The positive indication suggests that as a farmer's exposure to farm credits increases, so does the profitability of beef cattle production. The availability of finances for agribusiness (commercial production) is captured by access to

farm credit [36]. Furthermore, credit facilitates the purchasing of inputs, extension trips, and other assistance programs for diversified business activities, hence enhancing profitability [36].

Furthermore, the abundance of grazing resources for cattle production is represented by pasture accessibility [36]. Because stocking rates are not limited in rural areas, farmers' perceptions of abundant grazing pastures motivate them to participate in production operations, enhancing farm operating efficiency [36]. In this study, however, ~~pasture availability harmed beef cattle performance.~~ This demonstrates that as the grazing pasture increases, the profit per cattle diminishes. As pastures ~~develop~~ in rural regions, farmers tend to rely only on grazing ~~pasture rather~~ than additional feeds, resulting in lesser profit per cattle. Farmers are encouraged to feed their cattle with palatable and nutritious feeds to improve beef cattle production efficiency.

Additionally, while selling contracts provide farmers with a guaranteed market at their storefront, ~~lowering marketing and trading costs as well as market risks~~. However, the results of the ~~study~~ revealed that participating in selling contracts was significantly ~~negatively~~ correlated with the profitability of beef cattle production and was statistically significant at the 5% level of significance. This implies that a farmer, as a weaker partner, is vulnerable to exploitation by the ~~company~~ [43]. If farmers have committed significant investment into resources related to the contract commodities which are perishable and not conducive to translation into perishable items on the farm, a company can take monopoly rent in the output market. In addition, other significant negative externalities which hinder profitability are the danger of farmers engaging in extra-contractual sales, particularly when the agreed price is set and the stock value at the time of the sale is greater than the agreed price [43]. ~~As a result,~~ selling contracts should be mutually beneficial (flexible and profitable) to increase profitability.

Table 4. Multiple linear regression estimates using ordinary least squares (OLS) on the factors influencing the profitability of beef cattle farming among smallholder beef cattle farmers N = 388).

Parameters (variables)	Coefficients (β)	Std. Error
Feed costs	-0.713*	0.000
Husbandry labour costs	0.205	0.001
Medicines(treatment) costs	0.072*	0.000
Sales and logistics costs	-0.148*	0.018
Education level	0.066*	0.010
Family size	0.072*	0.004
Land owned	-0.084*	0.054
Cattle herd size	-0.107*	0.001
Experience in farming	0.196*	0.002
Access to veterinary services	0.142	0.062
Access to credits	1.369*	0.179
Pasture availability	-0.215*	0.016
Selling contract	-1.015*	0.197
Marketing channels (market outlets)	0.002	0.010
Access to market information	0.013	0.053
**Constant**	**91.729***	**0.104**
**R Squared (R^2)**	**0.840(84.0%)**	
**Adjusted R squared (Adj.R^2)**	**0.833(83.3%)**	

Note: *Indicate statistical significance level at 5 % ($P < 0.05$).*

4. Conclusion

Generally, in Tajikistan, owning beef cattle is an important survival mechanism for smallholder farmers for alleviating poverty. Profit maximization is one of the primary goals of beef cattle farming enterprises for the long performance and sustainability. Therefore, beef cattle farming enterprises should indeed strive to maximize profit to excel and flourish in a free economy. Nevertheless, smallholder beef cattle farmers are notorious for inefficient profit maximization, therefore, this study was set to assess the economic benefits of beef cattle farming and its determinants among smallholder beef cattle farmers; specifically to (i) analyze the socioeconomic characteristics of smallholder beef cattle farmers and (ii) examine the profitability of beef cattle farming and its determinants among smallholder beef cattle farmers. Based on the Resources Based Views (RBV) theory, as shown in the findings, the majority of

farmers had a high level of literacy, as well as access to farm credits, market information, pasture, and veterinary services, which resulted in high profitability (gross margin) of beef cattle production. This signifies that beef cattle production is a feasible (profitable) business in the study area. However, the potential for increased profitability is significant if existing resources are efficiently coordinated and production expenses, notably feed and healthcare costs, are minimized. Feed expenses accounted for the largest share of operating expenditures, followed by health care costs, implying that the Livestock and Pasture Development Project I & II (LPDP I & II) undertaken in the research region had minimal influence on feed and health care costs reduction. Results further revealed that beef cattle production is pretty new between most farming households, necessitating the development of knowledge and expertise for increased efficiency. Thus, the government should develop additional measures for addressing concerns such as capacity building, suitable and freely ~~available pasture~~ as well as health management, to boost beef cattle profitability among smallholder beef cattle farmers in Tajikistan.

Author contribution

Conceptualization: Zhang Yuejie.

Data curation: Jobirov Farrukh.

Formal analysis: Jobirov Farrukh.

Funding acquisition: Zhang Yuejie.

Investigation: Jobirov Farrukh.

Methodology: Cornel Anyisile Kibona.

Project administration: Zhang Yuejie.

Project administration: Zhang Yuejie.

Resources: Jobirov Farrukh.

Software: Zhang Yuejie.

Supervision: Zhang Yuejie.

Validation: Cornel Anyisile Kibona.

Visualization: Zhang Yuejie.

Writing – original draft: Jobirov Farrukh.

Writing – review & editing: Cornel Anyisile Kibona.

Both authors read and approved the final manuscript.

Funding

This research is supported and funded by the National Beef Cattle Industrial Technology System and Industrial Economy Research Project under the Ministry of Agriculture in the People's Republic of China (PRC) (CARS-37).

Conflicts of Interest

The authors have declared that no conflict of interest exists.

References.

1. Bebe BO, Udo HMJ, Rowlands GJ, and Thorpe W. Smallholder dairy systems in Kenya highlands: breed preferences and breeding practices. Livestock Production Science, 2003, 82(2-3), pp. 117-127.

2. The Republic of Tajikistan. Livestock and Pasture Development Project –II. Detailed design report. Main report and appendices, 2015.

3. Moll HAJ. Costs and benefits of livestock systems and the role of the market and nonmarket relationships. Agricultural Economics, 2005, 32(2), pp. 181-193.

4. Randolph TF, Schelling E, Grace D, Nicholson CF, Leroy JL, Cole DC, and Ruel M. Role of livestock in human nutrition and health for poverty reduction in developing countries. Journal of animal science, 2007.

5. Upton M. The Role of Livestock in Economic Development and Poverty Reduction. PPLPI Working Paper No. 10, Pro-poor Livestock Policy Initiative (PPLPI), FAO, Rome, 2004.

6. United States Agency for International Development (USAID). Grazing Lands, Livestock and Climate Resilient Mitigation in Sub-Saharan Africa: The State of the Science. USAID, 2015.

7. Sedik D. The Feed-Livestock Nexus in Tajikistan: Livestock Development Policy in Transition. FAO Regional Office for Europe and Central Asia. Policy Studies on Rural Transition no. x, 2009.

8. World Bank. Priorities for Sustainable Growth: A strategy for agriculture sector development in Tajikistan (Technical Annex 3: Livestock Sector Review), 2006. http://siteresources.worldbank.org/INTTAJIKISTAN/Resources/TASS-F_R_V.pdf.

9. Mountain Societies Development Support Programme (MSDSP). Household Survey of GBAO, 2003. www.untj.org/library.

10. AgtCATtajikistan. Agricultural Technology Commercialization Assessment. United States Agency for International Development by the USAID Enabling Agricultural Trade (EAT) project, implemented by Fintrac Inc, 2014.

11. Mirzoev S. and Sobirzoda S. Leveraging SME Finance through Value Chains in Tajikistan. ADBI Working Paper 1020. Tokyo: Asian Development Bank Institute, 2019. Available: https://www.adb.org/publications/leveraging-sme-finance-through-value-chains-tajikistan.

12. Barney J. Firm resources and sustained competitive advantage. Journal of Management, 1991, 17(1), 99–120.

13. Yazdanfar D. Profitability determinants among micro-firms: Evidence from Swedish data. International Journal of Managerial Finance, 2013, 9(2), 151–160.

14. Schmidt FL and Hunter JE. Methods of meta-analysis: Correcting error and bias in research findings: Sage publications, 2014.

15. Barney JB. Resource-based theories of competitive advantage: A ten-year retrospective on the resource-based view. Journal of Management, 2001, 27(6), 643–650.

16. Slater SF and Olson EM. A fresh look at industry and market analysis. Business Horizons, 2002, 45(1), 15–22.

17. Singh AS and Masuku MB. Sampling techniques & determination of sample size in applied statistics research: An overview. Int. J. Econ. Commer. Manag. 2014, 2, 1–22.

18. Kibona CA and Yuejie Z. Factors that influence market participation among traditional beef cattle farmers in the Meatu District of Simiyu Region, Tanzania. PLoS ONE. 2021, 16(4): e0248576. Available: https://doi.org/10.1371/journal. pone.0248576.

19. Yamane T. Statistics: An Introductory Analysis. 2nd Edn., Harper and Row, New York, 1967, Pages: 919.

20. Kibona CA and Yuejie Z. Examining Profitability, Viability, and Commercialization Level of Beef Cattle Production among Pastoralists in the Simanjiro District of the Manyara Region. Asian Journal of Agricultural Extension, Economics & Sociology, 2021, 39(2): 141-153; DOI: 10.9734/AJAEES/2021/v39i230539.

21. Mlote SN, Mdoe NS, Isinika AC, and Mtenga LA. Profitability analysis of small-scale beef cattle fattening in the Lake Zone in Tanzania. Journal of Agricultural Economics and Development, 2013, Vol. 2(5), pp. 203-216. Available online at http://academeresearchjournals.org/journal/jaed.

22. Maree JG. First steps in research. Van Schaik Publishers, Pretoria, 2007.

23. Gujarati. Basic Econometrics, Fourth Edition. McGraw-Hill Companies, 2004, p. 982.

24. Wernerfelt B. A resource- based view of the firm. Strategic management journal, 1984, 5(2), 171–180.

25. Curran J, Jarvis R, Blackburn RA, and Black S. Networks and small firms: Constructs, methodological strategies, and some findings. International Small Business Journal, 1993, 11(2), 13–25.

26. Perry SE and Williams TH. Earnings management preceding management buyout offers. Journal of Accounting and Economics, 1994, 18(2), 157–179.

27. Jovanovic B. Selection and the Evolution of Industry. Econometrica: Journal of the Econometric Society, 1982, 649–670.

28. Claver E, Molina J, and Tarí J. Firm and Industry Effects on Firm Profitability: a Spanish Empirical Analysis. European Management Journal, 2002, 20(3), 321–328.

29. Esmael Y, Bekele A, and Ketema M. Determinants of the level of smallholder farmers' participation in potato sales in Kofele District, Oromia Region, Ethiopia. J. Agric. Sci. Res. 2016; 3, 23–30.

30. Dalie S, Wantasen E, Anis S, and Pangemanan SP. Profit analysis of traditional beef cattle farm in Minahasa regency, Indonesia. Online J. Anim. Feed Res. 2015; 5(3):90-94.

31. Mafimisebi TE, Bobola OM, and Mafimisebi OE. Fundamentals of Cattle Marketing in South West, Nigeria: Analysing Market Intermediaries, Price Formation and Yield

Performance. 4th International Conference of the African Association of Agricultural Economist, 2013, Pp. 24.

32. Nasiru M, Haruna U, and Garba A. Economics of Livestock Marketing in Gamawa Local Government Area, Bauchi State, Nigeria. 8th AFMA Congress, November 25-29, 2012, Nairobi, Kenya. No. 159412. African Farm Management Association (AFMA).

33. Okoruwa VO, Elijah S, Busari AA, and Iyayi EA. Studied Profitability of Beef Cattle Fattening in the Cosmopolitan City of Ibadan, Oyo State. Tropical Journal of Animal Science, 2012, Vol. 1. Pp. 187-195.

34. Nasiru M. Economic Analysis of Cattle Marketing in Two Agricultural Development Programme Zones of Kano State, Nigeria". Ph.D. Thesis, Agricultural Economics, and Extension Programme, School of Agriculture, AbubakarTafawaBalewa University Bauchi, Nigeria, 2008.

35. Suleiman M, Isiaka M, and Ishaku A. Socioeconomic Factors Influencing Profitability of Cattle Marketing in Gombe Metropolis, Nigeria. International Journal of Scientific and Technology Research, 2013, Volume 2, No.12. Pp. 288-292.

36. Dlamin SI, Huang WC. A Double Hurdle Estimation of Sales Decisions by Smallholder Beef Cattle Farmers in Eswatini. Sustainability, 2019; 11:5185. DOI: 10.3390/su11195185.

37. Kiwanuka RN and Machethe C. Determinants of smallholder farmers' participation in Zambian dairy sector's interlocked contractual arrangements. J. Sustain. Dev. 2016, 9, 230–245.

38. Koknaroglu H, Loy DD, PAS DE, Wilson MP, Hoffman PAS, and Lawrence JD. Factors Affecting Beef Cattle Performance and Profitability. Applied Animal Science, 2005, https://doi.org/10.15232/S1080-7446 (15)31220-1; P286-296.

39. Kennedy PL, Harrison RW, and Piedra MA. Analyzing Agribusiness competitiveness: the case of United States Sugar Industry. International Food and Agribusiness Review, 1998, 1(2): 245-253.

40. Afolabi JA. Evaluation of Poultry Egg Marketing in South-Western Nigeria, International Journal of Poultry Science, 2007, Vol. 6. No. 5. Pp. 362-366.

41. Kgosikoma K and Malope P. Determinants of market participation and the institutional constraints: A case study of Kweneng West, Botswana. J. Agric. Ext. Rural Dev. 2016; 8, 178–186.

42. Kefyalew G. Analysis of Smallholder Farmer's Participation in Production and Marketing of Export Potential Crops: The Case of Sesame in Diga District, East Wollega

Zone of Oromia Regional State. Master's Thesis, Addis Ababa University, Addis Ababa, Ethiopia, 2012.

43. Birthal PS. Linking smallholder livestock producers to markets: Issues and Approaches. Keynote paper presented at the 67th Annual Conference of the Indian Society of Agricultural Economics at Bankers Institute of Rural Development, Lucknow, India, 2007.
